# Typhoid Fever: A Reduction and a Resurgence

**DOI:** 10.4269/ajtmh.23-0286

**Published:** 2023-06-12

**Authors:** James E. Meiring, Peter I. Johnston

**Affiliations:** ^1^ Department of Infection, Immunity and Cardiovascular Disease, University of Sheffield, Sheffield, United Kingdom;; ^2^ Institute of Infection, Veterinary and Ecological Sciences, University of Liverpool, Liverpool, United Kingdom;; ^3^ Malawi Liverpool Wellcome Research Programme, Blantyre, Malawi

Several population-based studies have recently published data on the incidence of typhoid fever.^1^^–^^4^ These studies redress gaps in our knowledge of disease burden in specific locations and contribute to improved estimates of the global burden of typhoid fever.^5^ These data are increasingly important to decision-makers in typhoid-endemic countries as they consider implementing typhoid conjugate vaccine (TCV) programs and apply for GAVI funding.^6^

Although typhoid incidence estimates are informative, we should be cautious in generalizing their findings beyond the period during which data were collected. Typhoid incidence is dynamic, with increases following the introduction of new strains with increased antimicrobial resistance,^7^^,^^8^ and reductions following the introduction of disease control interventions.^9^ Long-term incidence data from single-sites is rare, but can provide crucial insights into how various factors influence rates of typhoid fever.^10^ This is why the study by Ng’eno et al., published in a recent issue of the *American Journal of Tropical Medicine and Hygiene*, is so relevant.^11^

Ng’eno et al. provide a decade of typhoid incidence data from the informal Kenyan settlement of Kibera. The authors report crude incidence rates ranging from 144 to 233 per 100,000 person-years of observation (pyo) between 2010 and 2012. This was followed by a decline in typhoid incidence from 2013 to 2017. Cases rebounded in the final two years of surveillance, reaching 130 per 100,000 pyo in 2019. The authors speculate that government-instituted improvements in the supply of clean water (introduced in 2010) might explain the reduced incidence rates observed between 2013 and 2017. The subsequent resurgence in cases from 2019 serves an important reminder: in a world facing climate change and extreme weather events, population growth, and urbanization, typhoid fever remains at risk of resurging in populations without typhoid-specific immunity throughout south Asia and Africa.

The other benefit of this longitudinal study is that changes in antimicrobial resistance (AMR) patterns can be observed over time. The authors report that more than 70% of *Salmonella enterica* serovar Typhi (*S.* Typhi) isolates recovered from blood cultures were multidrug resistant (MDR) (resistant to ampicillin, chloramphenicol, and co-trimoxazole). While the proportion of MDR isolates remained relatively stable over time, nonsusceptibility to ciprofloxacin (CipNS) increased during the study period.

AMR patterns seen in this study are consistent with those seen in other studies from Africa^12^ and the findings from a large analysis of global typhoid genomes produced by Carey et al.^13^ Their analysis of almost 13,000 *S.* Typhi genomes showed that MDR *S.* Typhi remains widespread in Kenya and Malawi, although it has become less prevalent in Nigeria, India, Nepal, and Bangladesh.^13^ Longitudinal datasets allow us to develop hypotheses about how resistance patterns emerge and to monitor interventions. 19% of the MDR isolates in Kenya showed chromosomal integration of the MDR transposon, which carries less of a fitness-cost than plasmid-mediated MDR determinants.^13^

Similarly, the authors draw timely attention to the increased proportion of CipNS seen over time in Kibera (from 33% in 2014–41% in 2019). This corresponds with findings from Carey and colleagues’ genomic analysis, where the proportion of CipNS *S.* Typhi isolates in Kenya increased from 20% in 2012–65% in 2016.^13^ Longitudinal changes in resistance patterns may result in future outbreaks of disease. Multiple introductions of the MDR-associated H58 lineage of *S.*Typhi were associated with large-scale outbreaks in South Asia and Africa.^14^ Typhoid outbreaks in Pakistan’s Sindh province heralded the emergence of extensively drug resistant (XDR) *S.* Typhi^15^ (defined as MDR, with additional resistance to fluoroquinolones and third-generation cephalosporins). Several XDR variants are now circulating within Pakistan.^16^ The threat of XDR *S.* Typhi emerging in Africa means that long term sentinel-site surveillance, such as that presented by Ng’eno et al., is vital. Data such as these will also inform treatment guidelines, especially in settings where high typhoid incidence coincides with a lack of access to blood culture and antimicrobial susceptibility testing facilities.^17^

Typhoid fever is a disease that reflects global inequalities. In industrialised nations typhoid fever all but disappeared following improvements in sanitation during the early twentieth century.^18^ In contrast, typhoid remains among the commonest causes of fever and bloodstream infection throughout South Asia, Africa, and parts of Oceania. However, with new data on the efficacy of TCV the global community now has the tools to address this global inequality. Randomized controlled trials from Malawi,^19^ Nepal^20^^,^^21^ and Bangladesh^22^ all demonstrated protective efficacy of almost 80% and above after a single dose of TCV in children between 6 months and 15 years of age. In addition, effectiveness studies from Pakistan and India showed high levels of protection from a single dose of TCV, including against XDR *S.* Typhi.^23^^,^^24^ As typhoid endemic countries begin to incorporate TCV into their national immunization programs, studies that report longitudinal incidence data – such as this one from Ng’eno et al. – will be essential to understand the impact of vaccination on typhoid incidence and *S.* Typhi resistance.
